# Psychometric properties of the Nursing Home Survey on Patient Safety Culture in Norwegian nursing homes

**DOI:** 10.1186/s12913-016-1706-x

**Published:** 2016-08-27

**Authors:** Kathrine Cappelen, Karina Aase, Marianne Storm, Jørn Hetland, Anette Harris

**Affiliations:** 1 Centre for Caring Research South, Faculty of Health and Social Sciences, University College of Southeast Norway, Porsgrunn, Norway; 2 Department of Health Studies, Faculty of Social Sciences, University of Stavanger, Stavanger, Norway; 3 Department of Psychosocial Science, Faculty of Psychology, University of Bergen, Bergen, Norway

**Keywords:** Nursing homes, Patient safety, Perception, Quality improvement, Safety culture, Psychometrics

## Abstract

**Background:**

Developing a culture where staff are actively aware of how to prevent adverse events is a challenge. The use of survey tools to assess the status of patient safety culture seems to be acceptable as an early step in improving patient safety. The Nursing Home Survey on Patient Safety Culture (NHSOPSC) includes 12 dimensions and is specifically developed for nursing homes. In this study, we describe a Norwegian version of the NHSOPSC and assess its psychometric properties when tested on a sample of healthcare staff in nursing homes.

**Methods:**

The NHSOPSC was translated into Norwegian and pilot tested before being distributed to 12 nursing homes in Norway. Of the 671 healthcare staff invited, 466 (69 %) answered the questionnaire. SPSS 23.0 was used for descriptive data analysis and estimating internal consistency (Cronbach’s alpha). The dimensional structure of the questionnaire was tested by confirmatory factor analysis (CFA) using Mplus (version 7.2).

**Results:**

The CFA testing of the original 12-factor solution suggested that some modifications were needed because of the high correlations between three of the latent factors. A subsequent analysis resulted in a final ten-factor solution. The final model showed acceptable fit to the data (root mean square error of approximation = 0.060, 90 % confidence interval: 0.057–0.063, comparative fit index = 0.934, Tucker-Lewis index = 0.926, *χ*^2^ = 2058.33, df = 765, *p* < 0.001) and acceptable factor loadings ranging from 0.402 to 0.891. Moreover, moderate-to-strong correlations ranging from 0.455 to 0.812 were found between the ten latent factors. Finally, moderate-to-high correlations were found between the ten latent factors and an overall rating of patient safety in the nursing home.

**Conclusions:**

Factor analysis indicated that a modified ten-factor model fitted the data set in a Norwegian community healthcare context with acceptable goodness-of-fit values and could be recommended as a useful tool to assess staff perceptions of patient safety issues in Norwegian nursing homes.

**Electronic supplementary material:**

The online version of this article (doi:10.1186/s12913-016-1706-x) contains supplementary material, which is available to authorized users.

## Background

Proven methods and useful tools for measuring quality of care and patient safety in nursing homes appear to be lacking, although studies have shown that patient safety culture is poorly developed and patients may be at risk of harm [1]. Adverse events also seem to be common in long-term care, including nursing homes [2, 3]. One of the most discussed areas within the field of patient safety is how to develop a culture where staff are actively aware of the potential for adverse events [3–6].

Safety culture may be considered part of the organizational culture; it creates norms and influences staff attitudes and behavior. The Agency for Healthcare Research and Quality (AHRQ) refers to the following definition of safety culture, which is also applicable to nursing homes: “The safety culture of an organization is the product of individual and group values, attitudes, perceptions, competencies, and patterns of behavior that determine the commitment to, and the style and proficiency of, an organization’s health and safety management. Organizations with a positive safety culture are characterized by communications founded on mutual trust, by shared perceptions of the importance of safety, and by confidence in the efficacy of preventive measures.” [7] p. 23.

Safety culture is a complex phenomenon, which makes it difficult to operationalize [4, 8]. An extensive range of safety culture properties have been identified and organized into dimensions or so called subcultures, such as leadership, teamwork, evidence-based patient care, communication, learning, just culture, and patient centered care, which form a safety culture framework [8]. These dimensions may represent ways of conceptualizing safety culture. Halligan and Zecevic [4] found that the most frequently cited dimensions were leadership commitment to safety, open communication founded on trust, organizational learning, a nonpunitive approach to adverse event reporting and analysis, teamwork, and shared belief in the importance of safety [4].

Studies from both hospitals and nursing homes point at the association between patient safety culture and clinical outcomes such as safe care [1, 3, 9, 10]. Major efforts have been carried out to improve patient safety, such as campaigns, programs and adverse event reporting systems [11]. In 2011, the Norwegian Ministry of Health and Care Services launched the campaign, “In Safe Hands,” which continued as a national program for patient safety with increased attention on primary care, including nursing homes. Assessing patient safety culture is a major strategy in the ongoing program.

Several validated instruments for mapping safety culture in healthcare organizations are accessible, including the Safety Attitude Questionnaire and Hospital Survey of Patient Safety Culture (HSOPSC) [6, 12, 13]. In 2004, the AHRQ released the HSOPSC, and it has since been translated and tested in different countries [14]. Although it was originally developed in the United States, 44 different European studies conducted in 20 different countries have used the HSOPSC [15]. A Swedish version of the HSOPSC has been tested on a large sample in both hospitals and primary healthcare [16].

In 2008, in a response to interest shown by nursing homes in an instrument similar to the HSOPSC, the AHRQ released the Nursing Home Survey on Patient Safety Culture (NHSOPSC). The NHSOPSC is designed especially for nursing home staff and explores their perceptions of patient safety culture [17]. According to the AHRQ, NHSOPSC can be used in the following ways: “As a diagnostic tool to assess the status of patient safety culture in a nursing home, as an intervention to raise staff awareness about patient safety issues, as a mechanism to evaluate the impact of patient safety culture improvement initiatives, and as a way to track changes in patient safety culture over time.” [17] p. 1.

The NHSOPSC is in line with Schein’s definition of organizational culture defined as: “(a) a pattern of basic assumptions, (b) invented, discovered, or developed by a given group, (c) as it learns to cope with its problems of external adaptation and internal integration, (d) that has worked well enough to be considered valid and, therefore (e) is to be taught to new members as the (f) correct way to perceive, think, and feel in relation to those problems.” [18] p. 123. The central core is the basic assumptions and shared perceptions, primarily unconscious and representing learned responses based on experiences. Over time, this may develop into a common practice, “this is how we do it here”.

Even though the NHSOPSC has been available for some years, its use in Europe is still limited. The only available validation study in a European context is a Swiss version (translated into German) based on data from nine nursing homes (*n* = 477). The Swiss version gave a nine-dimensional fit as opposed to the 12 original dimensions. The study concluded that the survey’s dimensionality needed further clarification, mainly to distinguish items addressing the unit-level from those at the facility level [19]. Hence, there is a need for further studies to test the original version of NHSOPSC. The Swiss study applied exploratory factor analyses, often resulting in a reduction in number of factors due to the use of a data reduction method.

The aim of this study is therefore to test the psychometric properties of the Norwegian version of the original NHSOPSC by asking for staff perceptions of patient safety culture in a sample of Norwegian nursing homes. Based on previous research and the original structures and theoretical domains our hypothesis was that the original factor structure of the NHSOPSC could be replicated in a Norwegian context [1, 2, 17].

## Methods

### Design, setting, and recruitment

The study was designed as a cross-sectional survey examining staff perceptions of patient safety in nursing homes using a Norwegian version of the NHSOPSC. The study has been reported according to the STROBE checklist for cross-sectional studies (Additional file 1).

Healthcare staff from 12 different nursing homes in southern and western Norway, including urban and rural districts, received the survey. The nursing homes varied in size and organization. Norwegian nursing homes offer advanced care such as long- and short-term care, subacute and acute care, rehabilitation, specialized care for patients with dementia and cognitive impairments, and palliative care.

Inclusion criteria were staff, defined as healthcare workers with a minimum of 30 % part-time position and experience with the nursing home policies and day-to-day activities. Staff working in both nursing homes and assisted living or home care had to work at least a 30 % part-time position in the nursing home. All respondents had to be able to read and understand Norwegian.

### Data collection

A total of 671 questionnaires were distributed as paper versions to 12 nursing homes in southern and western Norway. An informative letter presenting the study with a return envelope accompanied the questionnaires. The survey was conducted in June–September, 2013.

### Questionnaire

The NHSOPSC includes 43 survey items measuring 12 dimensions (Table 1). The questionnaire is divided into four sections (A, B, C, and D) in addition to overall ratings of the nursing home, and background variables [17].

**Table 1:** The original patient safety culture dimensions of the NHSOPSC used in the Norwegian nursing home study

Patient safety culture dimensions	Items
1. Teamwork	4
2. Staffing	4
3. Compliance with procedures	3
4. Training and skills	3
5. Nonpunitive response to mistakes	4
6. Handoffs	5
7. Feedback and communication about incidents	4
8. Communication openness	3
9. Supervisor expectations and actions promoting patient safety	3
10. Overall perceptions of patient safety	3
11. Management support for patient safety	3
12. Organizational learning	4

Nine of the 12 dimensions in NHSOPSC are similar to the HSOPSC, but the included items are different. All items in NHSOPSC are rated on Likert-type scales from 1 to 5 with verbal anchors. “Does not apply or don’t know” was included as a response category. Overall rating questions considered as outcomes consist of a single statement, “I would tell friends that this is a safe nursing home for their family” (Yes, Maybe, No) and a graded overall rating, “Please give this nursing home an overall rating on patient safety” (scale from 1 to 5).

### Translation procedures and content validity

The US version of the NHSOPSC was first translated to Norwegian by a translation team, and then back-translated by an independent and experienced translator fluent in English and Norwegian according to recommended guidelines [20]. The translation team members were fluent in both languages and included an occupational therapist with a background in community healthcare and nursing homes and experienced in translating surveys, a research assistant with experience in surveys and epidemiology, an assistant professor and researcher within elderly care, and a coordinator. The draft translation was pretested in a group of healthcare staff working in nursing homes and community healthcare representing different professions such as nurses responsible for quality work, an apprentice, nurse managers, a medical doctor, and an assistant (*n* = 7). The pretest emphasized whether or not the items were relevant and understandable for the users. The final review was accepted by an external researcher who had also translated the hospital version (HSOPSC) of the instrument into Norwegian [21].

The Norwegian version retained 43 items. One item, B3, “We have all the information we need when residents are transferred from hospital,” was split into B3a, “We get the medical information when patients are transferred from hospital” and B3b, “We get the nursing report when patients are transferred from hospital.” The AHRQ guidelines [20] were adjusted to a Norwegian healthcare context and the role of nursing homes. Consequently, the concept of resident safety was replaced by patient safety, which is in line with the legal definition of a patient in Norway [22]. Furthermore, in the Norwegian version, the focus is on the “the nursing home as a whole” referred to as the facility level, using terms like “our nursing home” and “your nursing home.” The sample of nursing homes differs in both size and organization.

### Statistical analysis

#### Response rate and variability

Response rate and variability were examined with frequency analysis. Response variability was considered low when 90 % or more of the respondents chose the answer “agree/strongly agree” or “most of the time/always”. Chi-square for independency was used to examine if the number of “does not apply or don’t know” and number of missing values differed significantly based on respondents’ background variables. The analyses were performed with SPSS (version 23.0; IBM SPSS, Armonk, NY). *P*-value < 0.05 was considered significant.

### Confirmatory factor analysis (internal structure)

Structural equation modeling was used to test how the original 12-factor solution fitted the Norwegian data. The means and variance adjusted weighted least square estimator appropriate for ordinal data were used. Missing data, including the response option “does not apply or don’t know”, were handled by pairwise deletion, which is the default when using this estimator. The best-fit model was assessed with the comparative fit index (CFI) [23], the Tucker-Lewis index (TLI) [24] and the root mean square error of approximation (RMSEA) [25]). CFI level above 0.95, TLI level above 0.90, and a RMSEA value less than 0.060 is recommended as a good fit [26]. Factor loadings were expected to be above 0.30 [27].

### Internal consistency

Cronbach’s alpha coefficients were used to measure internal consistency of the NHSOPSC instrument and its dimensions. Homogeneity was considered good if the alpha values were between 0.70 and 0.90 and acceptable if the alpha values were >0.60 [15].

### Validity based on relation to other variables (overall rating questions)

The two overall rating questions had substantially different connotations; therefore, they were treated as complementary questions. The first question, “I would tell friends that this is a safe nursing home for their family,” was explored using descriptive statistics (n, %) in SPSS (version 23.0; IBM SPSS, Armonk, NY). The correlation between the second overall rating question “Please give this nursing home an overall rating on patient safety,” and the modified Norwegian ten-factor model was assessed in Mplus (version 7.2; Muthén and Muthén, Los Angeles, CA). *P*-value < 0.05 was considered significant.

## Results

A total of 671 participants were invited of which 466 (69 %) returned the Norwegian NHSOPSC questionnaire. The respondents were managers (*n* = 29), healthcare workers with a minimum of a bachelor degree (*n* = 181), healthcare workers with upper secondary school education (*n* = 226), assistants (*n* = 12), and others (*n* = 9). Furthermore, 70 % of the respondents worked more than 25 h per week. Background variables are presented in Table 2.

**Table 2:** Background variables of the respondents in the Norwegian nursing home study (*n* = 466)

Background variables	Number	Percent
Staff position or background (*n* = 457)		
Managers including leaders at first-line level	29	6.3
Healthcare workers with a minimum of bachelor degree	181	39.6
Healthcare workers, upper secondary school	226	49.5
Assistants	12	2.6
Others	9	2.0
Number and years in nursing home (*n* = 457)		
<1 year	29	6.3
1–5 years	114	24.9
6–10 years	105	23.0
11–15 years	105	23.0
16–20 years	44	9.6
>21 years	60	13.1
Work hours per week (*n* = 454)		
<15 h	7	1.5
16–24 h	121	26.7
25–35.5 h	253	55.7
>35.5 h	73	16.1
Work shift (most often) (*n* = 447)		
Daytime	303	67.8
Afternoon	85	19.0
Nighttime	59	13.2
Working directly with patients most of the time (*n* = 458)		
Yes	436	95.2
No	22	4.8

### Response rate and variability

The response rates per item and the number of “does not apply or don’t know” responses, and the number of missing responses are presented in Table 3. Missing values range from 3 to 36. The highest missing values were related to the items; “We receive medical information when patients are transferred from hospital” (B3a) and “We receive nursing report when patients are transferred from hospital” (B3b). The responses “does not apply or don’t know”, also treated as missing values in the analysis, range from 0 (0 %) to 88 (19 %).

**Table 3:** Dimensions/items with corresponding mean and standard deviation (SD), response rate (n %), responses to “Does not apply, Do not know” category, and missing values

Dimensions/Items	Mean (SD)	Response rate n (%)	Does not apply or don’t know	Missing
1. Teamwork				
A1. Staff in this nursing home treat each other with respect	4.31 (0.79)	463 (69 %)	0	3
A2. Staff support one another in this nursing home	4.18 (0.79)	459 (68 %)	0	7
A5. Staff feel like they are part of a team	4.10 (0.82)	463 (69 %)	0	3
A9. When someone gets really busy in this nursing home, other staff help out	3.71 (0.85)	459 (68 %)	1	6
2. Staffing				
A3. We have enough staff to handle the workload	2.97 (0.89)	456 (68 %)	3	7
A8 (R). Staff have to hurry because they have too much work to do	2.55 (0.92)	458 (68 %)	2	6
A16. Patients need are met during shift changes	3.80 (0.81)	450 (67 %)	9	7
A17 (R). It is hard to keep patients safe because so many staff quit their jobs	4.11 (0.85)	427 (64 %)	30	9
3. Compliance with procedures				
A4. Staff follow standard procedures to care for patients	4.00 (0.79)	455 (68 %)	6	5
A6 (R). Staff use shortcuts to get their work done faster	3.38 (0.92)	450 (67 %)	11	5
A14 (R). To make work easier, staff often ignore procedures	3.82 (0.81)	444 (66 %)	17	5
4. Training and skills				
A7. Staff get the training they need in this nursing home	3.63 (0.86)	460 (69 %)	3	3
A11. Staff have enough training on how to handle difficult patients	3.21 (0.86)	450 (67 %)	8	8
A13. Staff understand the training they get in this nursing home	3.89 (0.73)	437 (65 %)	20	9
5. Nonpunitive response to mistakes				
A10 (R). Staff are blamed when a patient is harmed	4.13 (0.77)	427 (63 %)	30	9
A12 (R). Staff are afraid to report their mistakes	3.76 (0.83)	435 (65 %)	26	5
A15. Staff are treated fairly when they make mistakes	3.95 (0.79)	417 (62 %)	40	9
A18. Staff feel safe reporting their mistakes	3.97 (0.74)	447 (67 %)	14	5
6. Handoffs				
B1. Staff are told what they need to know before taking care of a patient for the first time	4.01 (0.72)	458 (68 %)	4	4
B2. Staff are told when there is a change in a patients’ care plan	3.79 (0.78)	447 (67 %)	13	6
B3a. We have all the information we need when patients are transferred from the hospital (medical information)	4.09 (0.80)	386 (58 %)	44	36
B3b. We have all the information we need when patients are transferred from the hospital (nursing report)	4.24 (0.74)	381 (57 %)	55	30
B10. Staff are given all the information they need to take care of patients	4.24 (0.63)	458 (68 %)	1	7
7. Feedback and communication about incidents				
B4. When staff report something that could harm a patient, someone takes care of it	4.25 (0.69)	434 (65 %)	27	5
B5. In this nursing home, we talk about ways to keep patients from happening again	3.98 (0.78)	453 (68 %)	3	10
B6. Staff tell someone if they see something that might harm a patient	4.42 (0.58)	454 (68 %)	6	6
B8. In this nursing home, we discuss ways to keep patients safe from harm	4.07 (0.69)	450 (67 %)	6	10
8. Communication openness				
B7. Staff ideas and suggestions are valued in this nursing home	3.85 (0.74)	457 (68 %)	1	8
B9 (R). Staff opinions are ignored in this nursing home	3.82 (0.83)	448 (67 %)	8	10
B11. It is easy for staff to speak up about problems in this nursing home	3.90 (0.85)	446 (66 %)	12	8
9. Supervisor expectations and actions promoting patient safety				
C1. My supervisor listen to staff ideas and suggestions about patient safety	4.22 (0.76)	453 (68 %)	6	7
C2. My supervisor says a good word to staff who follow the right procedures	4.19 (0.78)	452 (67 %)	6	8
C3. My supervisor pays attention to patient safety problems in this nursing home	4.38 (0.66)	450 (67 %)	9	7
10. Overall perceptions of patient safety				
D1. Patients are well cared for in this nursing home	4.33 (0.73)	462 (69 %)	1	3
D6. This nursing home does a good job keeping patients safe	3.97 (0.64)	449 (67 %)	11	6
D8. This nursing home is a safe place for patients	4.30 (0.64)	461 (69 %)	1	4
11. Management support for patient safety				
D2. Management asks staff how the nursing home can improve patient safety	3.58 (0.96)	423 (63 %)	33	10
D7. Management listen to staff ideas and suggestions to improve patient safety	3.80 (0.83)	443 (66 %)	19	4
D9. Management often walks around the nursing home to check on patients care	2.96 (1.14)	412 (61 %)	45	9
12. Organizational learning				
D3 (R). This nursing home lets the same mistakes happen again and again	3.70 (0.85)	433 (65 %)	27	6
D4. It is easy to changes to improve patient safety in this nursing home	3.59 (0.79)	439 (65 %)	18	9
D5. This nursing home is always doing things to improve patient safety	3.79 (0.74)	444 (66 %)	18	4
D10. When this nursing home makes changes to improve patient safety, it checks to see if the changes worked	3.61 (0.84)	365 (54 %)	88	13

Item-response categories: Items A1–A18, items C1–C3, and items D1–D10: 1 = strongly disagree; 2 = disagree; 3 = neither agree nor disagree; 4 = agree; 5 = strongly agree; item B1–B11: 1 = never; 2 = rarely; 3 = sometimes; 4 = most of the time; and 5 = always

*R* reverse coded

The items in the NHSOPSC instrument are rated on scales from 1 to 5. The results showed that all five response alternatives had been used for 36 of the items, while the response alternatives 2–5 had been used for eight of the items: “Staff are told what they need to know before taking care of a patient for the first time” (B1), “We receive nursing report when patients are transferred from hospital (B3b), “When staff report something that could harm a patient, someone takes care of it” (B4), “Staff tell someone if they see something that may harm a patient” (B6), “Staff are given all the information they need to take care for patients” (B10), “This nursing home does a good job keeping patients safe” (D6), “This nursing home is a safe place for patients” (D8) and the overall rating question “Please give this nursing home an overall rating on patient safety” (E2).

The response variability was below 90 % for all items except “Staff tell someone if they see something that may harm a patient” (B6), “Staff are given all the information they need to take care for patients” (B10), “My supervisor pays attention to patient safety problems in this nursing home” (C3) and “This nursing home is a safe place for patients” (D8) in which the response variability was 96, 91, 93 and 91 % respectively.

Some items have a relatively high response rate to the “does not apply or don’t know” category. Results revealed that mainly staff with lower education, staff having worked less than a year or part time workers had missing responses or responded “does not apply or don’t know”. Significant differences in responses “does not apply or don’t know” according to the background variables are listed in Appendix A (Additional file 2) and significant differences in missing responses according to the background variables are listed in Appendix B (Additional file 3).

### Confirmatory factor analysis (internal structure)

In accordance with the expected dimensionality of the NHSOPSC instrument, we first tested a confirmatory factor analysis (CFA) with 12 latent factors and their respective indicators (*n* = 347). Factors were allowed to correlate in the model. However, the initial CFA showed a negative definite matrix for some of the latent factors. The analysis indicated that this was caused by an almost perfect correlation between the latent factors “Organizational learning,” “Overall perceptions of patient safety” (*r* = 0.94), and “Management support for patient safety” (*r* = 0.94). These high correlations indicate that these latent factors could be merged into one factor labeled “Management and organization learning.” The results were in accordance with a Swiss validation of the NHSOPSC instrument [19] and in accordance with the organizational structure in Norwegian nursing homes. Accordingly, we therefore tested a ten-factor model where the three factors were merged. The model fit was acceptable (RMSEA = 0.063, 90 % confidence interval [CI]: 0.060–0.066, CFI = 0.921, TLI = 0.912, *χ*^2^ = 2317.67, df = 815, *p* < 0.001). However, the standardized factor loadings for two of the indicators, B3a and B3b, were <0.40 (0.158 and 0.345, respectively). Therefore, we tested a modified model omitting these indicators from the respective factor (F6). The modified ten-factor model showed a better fit (RMSEA = 0.060, 90 % CI: 0.057–0.063, CFI = 0.934, TLI = 0.926, *χ*^2^ = 2058.33, df = 765, *p* < 0.001). In the final model, the factor loadings varied between 0.402 and 0.891 and loaded significantly (*p* < 0.001) on the latent factors for all indicators (Table 4). The correlations between the 10 latent factors were moderate to strong and varied between 0.455 and 0.812 (Table 5).

**Table 4:** Confirmatory factor analysis of the Norwegian NHSOPSC ten-factor model with corresponding factor loadings and reliability (Cronbach’s alpha) (*n* = 347)

Factors (Cronbach’s alpha) Items	Factor loading
F1 Teamwork (*α* = 0.79)	
A1	0.883
A2	0.885
A5	0.859
A9	0.538
F2 Staffing (*α* = 0.55)	
A3	0.556
A8 (R)	0.402
A16	0.653
A17 (R)	0.573
F3 Compliance with procedures (*α* = 0.58)	
A4	0.781
A6 (R)	0.447
A14 (R)	0.648
F4 Training and skills (*α* = 0.67)	
A7	0.737
A11	0.662
A13	0.715
F5 Nonpunitive response to mistakes (*α* = 0.65)	
A10 (R)	0.433
A12 (R)	0.558
A15	0.767
A.18	0.834
F6 Handoffs (*α* = 0.74)	
B1	0.758
B2	0.709
B10	0.826
F7 Feedback and communication about incidents (*α* = 0.74)	
B4	0.752
B5	0.675
B6	0.689
B8	0.800
F8 Communication openness (*α* = 0.74)	
B7	0.778
B9 (R)	0.673
B11	0.812
F9 Supervisor expectations and actions promoting patient safety (*α* = 0.84)	
C1	0.891
C2	0.865
C3	0.857
F10 Management and organizational learning (new factor) (*α* = 0.90)	
D1	0.733
D2	0.764
D3 (R)	0.705
D4	0.759
D5	0.816
D6	0.869
D7	0.818
D8	0.820
D9	0.589
D10	0.733

The new factor management and organizational learning includes “overall perception of safety,” “management support for patient safety,” and “organizational learning.”

Item-response categories: Items A1–A18, items C1–C3, and items D1–D10: 1 = strongly disagree; 2 = disagree; 3 = neither agree nor disagree; 4 = agree; 5 = strongly agree; item B1–B11: 1 = never; 2 = rarely; 3 = sometimes; 4 = most of the time; and 5 = always

*R* reverse coded items

**Table 5:** Correlation between the 10 latent factors in the Norwegian NHSOPSC measured with Mplus (*n* = 347). All *p*-values < 0.001

	F1	F2	F3	F4	F5	F6	F7	F8	F9
F1	1								
F2	.609	1							
F3	.642	.675	1						
F4	.675	.782	.758	1					
F5	.603	.664	.647	.663	1				
F6	.491	.634	.613	.755	.455	1			
F7	.535	.609	.614	.649	.595	.812	1		
F8	.685	.705	.635	.709	.715	.725	.784	1	
F9	.591	.510	.506	.602	.632	.521	.543	.770	1
F10	.593	.716	.650	.715	.553	.730	.751	.779	.627

F1 = Teamwork, F2 = Staffing, F3 = Compliance with procedures, F4 = Training and skills, F5 = Nonpunitive response to mistakes, F6 = Handoffs, F7 = Feedback and communication about incidents, F8 = Communication openness, F9 = Supervisor expectations and actions promoting patient safety, F10 = Management and organizational learning (new factor)

### Internal consistency

Results of the internal consistency analyses of the Norwegian NHSOPSC ten-factor model showed that eight of the ten factors reached an acceptable level of internal consistency with Cronbach’s alpha values >0.60. Two factors showed low values: “Staffing” (0.55) and “Compliance with procedures” (0.58) (Table 4).

### Validity based on relation to other variables (overall rating questions)

Correlation analyses performed in Mplus showed moderate-to-strong correlation between all factors in the modified ten-factor model and the overall rating question, “Please give this nursing home an overall rating of patient safety” (E2), with a range between 0.478 and 0.824 (Table 6).

**Table 6:** Correlations between the ten-factor model of the Norwegian NHSOPSC and the outcome measure item “Please give this nursing home an overall rating on patient safety” (E2) measured with Mplus (*n* = 337)

Factors	E2
F1 Teamwork	.498**
F2 Staffing	.664**
F3 Compliance with procedures	.527**
F4 Training and skills	.598**
F5 Nonpunitive response to mistakes	.520**
F6 Handoffs	.606**
F7 Feedback and communication about incidents	.608**
F8 Communication openness	.673**
F9 Supervisor expectations	.478**
F10 Management and organizational learning (new dimension)	.824**

**p < 0.01

The overall rating question, “I would tell friends that this is a safe nursing home for their family” (E1), documented that most of the respondents answered yes (86.6 %, *n* = 388), some answered maybe (12.7 %, *n* = 57), and only a few (0.7 %, *n* = 3) answered no.

## Discussion

Various safety culture tools are available to measure safety culture in healthcare organizations, among them surveys [28, 29].

We have described the results of a validation study using the Norwegian version of the NHSOPSC in a nursing home context. As such, it represents the first survey-based study of patient safety culture in Norwegian nursing homes. The response rate was satisfactory (69 %) compared with similar validation studies from Switzerland (66 %) [19], Norway (55 %) [21], and the United Kingdom (37 %) [14].

Regarding the number of factors, the CFA results from the Norwegian NHSOPSC study were in contrast to the original US version, but in accordance with the results from a Swiss study where the number of latent factors was reduced [17, 19]. The Swiss study concluded with nine factors, while our study concluded with ten factors. In the Swiss study, two factors “Overall perceptions of resident safety” and “Organizational Learning” were merged into one factor [19], while the same two factors and one additional factor “Management support for patient safety” were merged in the modified Norwegian model. Corresponding reductions of factors have been observed in similar validation studies of safety culture in hospitals where surveys are translated and tested in different contexts [14].

The distinction between facility and unit level within the nursing home was not applicable for the sample of nursing homes in this study because of differences in size and organizational models in the Norwegian setting. Therefore, the Norwegian version of the NHSOPSC focuses on the nursing home at facility level, referred to as “our nursing home,” “this nursing home,” and “your nursing home” in the questionnaire. Supervisors represent leaders at the lowest level conducting “day-to-day leadership” in nursing homes.

The items B3a and B3b did not contribute to the confirmed ten-factor model. During the translation and pretesting process, the original item, “We have all the information we need when residents are transferred from the hospital,” was split into two items related to medical information and nursing reports. By asking more specifically for information needed from the hospital (medical report, nursing report), these items no longer contributed to the model for measuring patient culture in nursing homes. This means that receiving medical information and nursing reports is still essential for patient safety but does not represent a cultural issue for nursing homes. Therefore, future studies should adhere to the original item B3 as one item. After removing the indicators B3a and B3b and reducing the number of factors, confirmatory analysis indicated that a ten-factor model best fitted the data set in a Norwegian community healthcare context with acceptable goodness-of-fit values.

In the Norwegian ten-factor model, the latent factors “Staffing” and “Compliance with procedures,” showed alpha values below 0.60. This might be explained by the number of items included in the latent factors besides some items showing a low loading. The factor “Compliance with procedures” included only three items, and one, “Staff use shortcuts to get their work done faster” (A6), had a low loading (0.447). Furthermore, the factor “Staffing” included four items, and one, “Staff have to hurry because they have too much work to do” (A8), had a low loading (0.402).

In this survey, both “Staffing” and “Compliance with procedures” are regarded as dimensions, although they are usually not among the most cited dimensions within the field of patient safety culture [4, 8]. Despite questionable internal consistency, we found no reason to remove these dimensions and related items from the questionnaire. These factors display important opinions and attitudes related to staffing and compliance with procedures that influence patient safety in nursing homes. Another argument for keeping the items in the instrument intact is to ensure the possibility for cross-country comparisons. The AHRQ has established comparative databases serving as important input for benchmarking and improvement efforts [30].

Finally, reliability measured with Cronbach’s alpha is not a recommended method when using latent factors [31]. A more appropriate method has been described by Raykov and Penev [32], but this method is only applicable for continuous variables so far.

Both the original NHSOPSC and the Norwegian version include only two overall rating questions (E1, E2). The correlations revealed a medium-to-large strength between the ten factors and the question, “Please give this nursing home an overall rating on resident safety” (E2). All ten factors reached statistical significance. The question, “I would tell friends that this is a safe nursing home for their family” (E1), revealed that more than 86 % of the respondents answered, “Yes”, indicating that it corresponds with the overall rating on patient safety. The Norwegian study shows relatively high scores. In comparison, an average of 76 % answered “Yes” to the same question in the US reference database including 263 nursing homes [30]. Presuming that there might be an association between safety culture and safe care for patients in nursing homes, more specific outcome measures related to adverse events would be of interest.

### Limitations

The prevalence of missing values in the current study was high; however, they were mainly linked to the category, “does not apply or don’t know”. We argue that this should be considered a valid answer by many of the respondents with lower education having worked less than a year or working part time, having in common that they may not yet hold sufficient information to be able to respond. For example the item, “When this nursing home makes changes to improve patient safety, it checks to see if the changes worked” (D10) revealed significant differences related to the background variable “number of years in the nursing home”.

Another limitation of the study is that we have not considered the possible cluster effect of unit versus facility levels in our analysis. However, a two-level confirmatory analysis was performed for the within-level factors with individuals (level 1) nested within units (level 2), accounting for the correlation of ratings within units (results not reported). The model showed poor fit, which was probably because of the relatively small sample size. However, the analysis showed interclass correlation variations between 0.005 and 0.0045, indicating that less than 5 % of the dimension variance was explained by unit/facility levels. Therefore, the unit/facility-level dimension was unessential in the Norwegian data set.

Challenges related to translation and use of surveys outside the geographical and healthcare context in which they are developed must be considered [14]. Organizational culture and the role of nursing homes in healthcare systems will differ across country-specific contexts because of current healthcare policies, reforms, and financial systems [33]. Nevertheless, questions related to patient safety cultures and staff awareness of adverse events will most likely have common denominators at a cross-national level. This requires some consistency regarding terminology and a minimum of shared items.

In this study staff’s opinions are treated as identical with staff’s perceptions and relate to how “reality” is interpreted. By asking for opinions, the survey also tries to map attitudes, but this raises several challenges related to the ability to measure attitudes. Several studies suggest the existence of an association between patient safety attitudes, staff behaviour and patient outcomes [1, 34]. However, the relationship between attitudes and behaviour seems to be complex and influenced by social factors such as management and staff support [35]. We have therefore limited our analysis to focus mainly on perceptions.

By conducting a NHSOPSC survey in nursing homes, patient safety issues will inevitably be put on the agenda. Therefore, the survey could be considered an intervention to raise awareness of patient safety issues among nursing home staff [17]. Discussing survey results may help nursing homes identify strengths and quality-improvement areas. Presenting the results may be a good starting point, but lasting changes depend on continuous work and survey follow-up [30].

## Conclusions

The study results indicated that a ten-factor model of the NHSOPSC instrument fitted the data set in a Norwegian community healthcare context with acceptable goodness-of-fit values. The NHSOPSC survey instrument seems to include the most-frequently theoretically cited dimensions related to safety culture as a concept. A validation study including a strategic selection of respondents from a larger sample of nursing homes will be required to assess safety culture at the unit/facility level.

## Additional files

Additional file 1: STROBE checklist for cross-sectional studies. (DOC 84 kb) Additional file 2: Appendix A including tables 7, 8, 9 and 10. Percentages of participants responding “does not apply or don’t know” according to staff position or background. (DOCX 18 kb) Additional file 3: Appendix B including tables 11, 12, 13 and 14. Percentages of “Missing” according to staff position or background. (DOCX 21 kb)

## Abbreviations

AHRQ: Agency for Healthcare Research and Quality
CFA: Confirmatory factor analysis
CFI: Comparative fit index
CI: Confidence interval
HSOPSC: Hospital Survey on Patient Safety Culture
NHSOPSC: Nursing Home Survey on Patient Safety Culture
RMSA: Root mean square error of approximation
TLI: Tucker-Lewis index
