# Supplementary material for: Psychometric properties of the Nursing Home Survey on Patient Safety Culture in Norwegian nursing homes
Source: BMC Health Serv Res. 2016 Aug 27;16(1):446. doi: 10.1186/s12913-016-1706-x (PMC5002111; doi:10.1186/s12913-016-1706-x)
Supplement: Additional file 2: — Appendix A including tables 7, 8, 9 and 10. Percentages of participants responding “does not apply or don’t know” according to staff position or background. (DOCX 18 kb) [file 12913_2016_1706_MOESM2_ESM.docx]

**Appendix A**

**Table 7** Percentages of participant that responded “does not apply or don’t know” according to staff position or background. Only significant differences are reported

|  | Managers including leaders at first-line level | Healthcare workers with a minimum of bachelor degree | Healthcare workers, upper secondary school | Assistants | Others | p-value |
| --- | --- | --- | --- | --- | --- | --- |
| B3a* | 7% | 4% | 14% | 30% | 29% | .001 |
| B3b* | 11% | 7% | 17% | 22% | 22% | .043 |
| B11 | 0% | 3% | 2% | 18% | 11% | .007 |
| D1 | 0% | 0% | 0% | 0% | 11% | .000 |

Notes: B3a: We receive medical information when patients are transferred from hospital; B3b: We receive nursing report when patients are transferred from hospital; B11: It is easy for staff to speak up about problems in this nursing home; D1: Patients are well cared for in this nursing home.

*Omitted in the final Norwegian 10-factor model.

**Table 8** Percentages of participants that responded “does not apply or don’t know” according to number of years in the nursing home. Only significant differences are reported

|  | < 1  year | 1-5  years | 6-10 years | 11-15 years | 16-20 years | > 21  years | p-value |
| --- | --- | --- | --- | --- | --- | --- | --- |
| A10 | 26% | 6% | 9% | 5% | 2% | 0% | .000 |
| A15 | 30% | 12% | 11% | 2% | 5% | 5% | .000 |
| D4 | 17% | 4% | 5% | 2% | 0 | 2% | .003 |
| D6 | 14% | 0% | 3% | 1% | 5% | 2% | .001 |
| D9 | 31% | 12% | 13% | 8% | 0% | 4% | .000 |
| D10 | 38% | 23% | 25% | 11% | 7% | 16% | .002 |

Notes: A10: Staff are blamed when a patient is harmed; A15: Staff are treated fairly when they make mistakes; D4: It is easy to make changes to improve patient safety in this nursing home; D6: This nursing home does a good job keeping patients safe; D9: Management often walk around the nursing home to check on patient care

D10: When this nursing home make changes to improve patient safety, it checks to see if changes worked.

**Table 9** Percentages of participants that responded “does not apply or don’t know” according to work hours per week. Only significant differences are reported

|  | < 15  hours | 16-24  hours | 25 – 35.5 hours | >35.5  hours | p-value |
| --- | --- | --- | --- | --- | --- |
| A4 | 14% | 1% | 0% | 4% | .002 |
| A11 | 0% | 0% | 1% | 7% | .003 |
| B1 | 14% | 2% | 0% | 1% | .001 |
| B3a* | 17% | 20% | 6% | 9% | .000 |
| B3b* | 33% | 20% | 8% | 15% | .004 |

Notes: A4: Staff follow standard procedures to take care for patients; A11: Staff have enough training on how to handle difficult patients; B1: Staff are told what they need to know before taking care of a patient for the first time; B3a: We receive medical information when patients are transferred from hospital; B3b: We receive nursing report when patients are transferred from hospital.

*Omitted in the final Norwegian 10-factor model.

**Table 10** Percentages of participants that responded “does not apply or don’t know” according to working time arrangement (most often). Only significant differences are reported

|  | Daytime | Afternoon | Nighttime | p-value |
| --- | --- | --- | --- | --- |
| A9 | 0% | 0% | 2% | .040 |
| A14 | 2% | 6% | 9% | .022 |
| D9 | 7% | 12% | 21% | .005 |

Notes: A9: When someone gets really busy in this nursing home, other staff help out; A14: To make work easier, staff often ignore procedures; D9: Management often walk around the nursing home to check on patient care.
