# Supplementary material for: Psychometric properties of the Nursing Home Survey on Patient Safety Culture in Norwegian nursing homes
Source: BMC Health Serv Res. 2016 Aug 27;16(1):446. doi: 10.1186/s12913-016-1706-x (PMC5002111; doi:10.1186/s12913-016-1706-x)
Supplement: Additional file 3: — Appendix B including tables 11, 12, 13 and 14. Percentages of “Missing” according to staff position or background. (DOCX 21 kb) [file 12913_2016_1706_MOESM3_ESM.docx]

**Appendix B**

**Table 11** Percentages of “Missing” according to staff position or background.

Only significant differences are reported

|  | Managers including leaders at first-line level | Healthcare workers with a minimum of bachelor degree | Healthcare workers, upper secondary school | Assistants | Others | p-value |
| --- | --- | --- | --- | --- | --- | --- |
| B3a* | 7% | 5% | 9% | 17% | 22% | .003 |
| B3b* | 7% | 4% | 8% | 25% | 0% | .016 |
| B5 | 0% | 1% | 2% | 8% | 22% | .003 |
| B8 | 0% | 2% | 2% | 17% | 0% | .028 |
| B11 | 0% | 1% | 2% | 8% | 0% | .021 |
| D1 | 0% | 1% | 0% | 0% | 0% | .000 |

Notes: B3a: We receive medical report when patients are transferred from hospital; B3b: We receive nursing report when patients are transferred from hospital; B5: In this nursing home, we talk about ways to keep incidents from happening again; B8: In this nursing home, we discuss ways to keep patients safe from harm; B11: It is easy for staff to speak up about problems in this nursing home; D1: Patients are well cared for in this nursing home.

*Omitted in the final Norwegian 10-factor model.

**Table 12** Percentages of “Missing” according to number of years in the nursing home.

Only significant differences are reported

|  | < 1  year | 1-5  years | 6-10 years | 11-15 years | 16-20 years | >21  years | p-value |
| --- | --- | --- | --- | --- | --- | --- | --- |
| A4 | 7% | 0% | 1% | 0% | 5% | 0% | .014 |
| A7 | 3% | 0% | 0% | 0% | 5% | 0% | .029 |
| A8 | 7% | 0% | 1% | 0% | 2% | 3% | .027 |
| A9 | 7% | 0% | 0% | 1% | 5% | 2% | .027 |
| A10 | 7% | 4% | 1% | 0% | 2% | 2% | .001 |
| A12 | 7% | 0% | 1% | 0% | 2% | 2% | .020 |
| A14 | 7% | 0% | 1% | 0% | 2% | 2% | .011 |
| A15 | 7% | 1% | 2% | 1% | 2% | 3% | .001 |
| A18 | 7% | 0% | 1% | 0% | 2% | 2% | .012 |
| B1 | 7% | 0% | 0% | 0% | 2% | 2% | .036 |
| D2 | 3% | 1% | 0% | 1% | 2% | 8% | .021 |
| D6 | 3% | 1% | 1% | 0% | 0% | 3% | .003 |
| D9 | 0% | 1% | 2% | 1% | 2% | 5% | .001 |

Notes: A4: Staff follow standard procedures to care for patients; A7: Staff get the training they need in this nursing home; A8: Staff have to hurry because they have to much work to do; A9: When someone gets really busy in this nursing home, other staff help out; A10: Staff are blamed when a patient is harmed; A12: Staff are afraid to report their mistakes; A14: To make work easier, staff often ignore procedures; A15: Staff are treated fairly when they make mistakes; A18: Staff feel safe reporting their mistakes; B1: Staff are told what they need to know before taking care of a patient for the first time; D2: Management asks staff how the nursing home can improve patient safety; D6: This nursing home does a good job keeping patients safe; D9: Management often walks around the nursing home to check on patient care.

**Table 13** Percentages of “Missing” according to work hours per week.

Only significant differences are reported

|  | < 15 hours | 16-24 hours | 25 – 35.5 hours | >35.5 hours | p-value |
| --- | --- | --- | --- | --- | --- |
| A4 | 0% | 2% | 0% | 3% | .005 |
| A11 | 0% | 2% | 2% | 3% | .026 |
| B1 | 0% | 2% | 0% | 1% | .003 |
| B3a* | 14% | 10% | 7% | 7% | .003 |
| B3b* | 14% | 7% | 6% | 7% | .034 |
| B5 | 0% | 5% | 0% | 1% | .017 |
| C2 | 0% | 5% | 0% | 0% | .005 |

Notes: A4: Staff follow standard procedures to care for patients; A11: Staff have enough training on how to handle difficult patients; B1: Staff are told what they need to know before taking care of a patient for the first time B3a: We receive medical report when patients are transferred from hospital; B3b: We receive nursing report when patients are transferred from hospital; B5: In this nursing home, we talk about ways to keep incidents from happening again; C2: My supervisor says a good word to staff who follow the right procedures.

*Omitted in the final Norwegian 10-factor model.

**Table 14** Percentages of “Missing” according to working time arrangement (most often).

Only significant differences are reported

|  | Daytime | Afternoon | Nighttime | p-value |
| --- | --- | --- | --- | --- |
| B3a* | 5% | 15% | 7% | .009 |
| D9 | 2% | 0% | 6% | .044 |

Notes: B3a: We receive medical report when patients are transferred from hospital; D9: Management often walks around the nursing home to check on patient safety.

*Omitted in the final Norwegian 10-factor model.
